# Intrasexual competition underlies sexual selection on male breeding coloration in the orangethroat darter, *Etheostoma spectabile*

**DOI:** 10.1002/ece3.2136

**Published:** 2016-04-20

**Authors:** Muchu Zhou, Rebecca C. Fuller

**Affiliations:** ^1^ Department of Animal Biology University of Illinois at Urbana‐Champaign Champaign Illinois 61820

**Keywords:** Darter, male coloration, male competition, sexual selection, visual signaling

## Abstract

Elaborate, sexually dimorphic traits are widely thought to evolve under sexual selection through female preference, male–male competition, or both. The orangethroat darter (*Etheostoma spectabile*) is a sexually dichromatic fish in which females exhibit no preferences for male size or coloration. We tested whether these traits affect individual reproductive success in *E. spectabile* when multiple males are allowed to freely compete for a female. The quality and quantity of male coloration were associated with greater success in maintaining access to the female and in spawning as the primary male (first male to participate). On the other hand, sneaking behavior showed little correlation with coloration. Male breeding coloration in *E. spectabile* may therefore demonstrate how intrasexual competition can be a predominant factor underlying the evolution of male ornaments.

## Introduction

Sexual selection theory, as originated by Darwin (1871), proposes that conspicuous male traits can evolve if they improve mating success through attractiveness to females, usefulness in competition with other males, or both. The notion that intrasexual contests can select for large body size or weaponry is fairly uncontroversial. On the other hand, male ornaments such as bright colors have traditionally been categorized as targets of female choice, and the effect of male–male competition on their evolution is less understood (Andersson 1994). Such ornaments though are involved in male–male competition across a wide range of taxa, as attested by numerous studies (e.g., Ligon et al. 1990; Mateos and Carranza 1997; Benson and Basolo 2006; Bajer et al. 2011; Crothers et al. 2011; Baird et al. 2013).

Conspicuous male traits may be favored through intrasexual selection if they enable males to remotely evaluate rivals and avoid the cost of fighting unnecessary battles with mismatched opponents (Maynard Smith and Parker 1976; Rohwer 1982; Maynard Smith and Brown 1986). In this capacity, male ornaments should communicate relevant attributes such as size, condition, or fighting ability (Parker 1974; Zahavi 1977, 1981). A well‐studied example is melanin‐ or carotenoid‐based badges of status, which are kept “honest” by physiological limitations and/or social mechanisms (Johnstone and Norris 1993; Jawor and Breitwisch 2003; Whiting et al. 2003). In rare cases, male–male competition has been found to drive color evolution independent of female choice (Grether 1996). These data suggest that male–male competition can select for the same types of ornaments as female choice and that the latter should not simply be presumed as the underlying cause of male ornamentation. Indeed, male–male competition has been proposed to be more effective than female preferences in promoting the elaboration of male ornaments: as variation in an honest signal of male quality need not rely on genetic variation and can be maintained in the face of ongoing selection, the signal should thus remain useful over time and persist in the population (Berglund et al. 1996).

Sexual dichromatism is prevalent in the darters (Percidae: Etheostomatinae), a species‐rich clade of North American freshwater fishes. The spectacular and diverse male coloration found in many darter species has long been considered to result from sexual selection (Reeves 1907; Mendelson 2003) and has sparked interest in the role that male coloration may have played in facilitating darter speciation (Martin and Mendelson 2013; Williams et al. 2013). Surprisingly, little empirical data exist on the mechanism by which sexual selection operates on male coloration in darters, or on whether coloration affects male reproductive success at all. In fact, Pyron (1995) and Fuller (2003) did not find the colorfulness of male darters to be predictive of male reproductive success.

Here, we investigate whether male coloration in the orangethroat darter (*Etheostoma spectabile*) (Agassiz, 1854) is under sexual selection via male–male competition. During the breeding season, male *E. spectabile* closely follow females while attempting to drive away rival males. When the female is ready to spawn, she buries herself shallowly in gravel and waits for a male to arrive. Females may spawn multiple times in short succession, and neither sex exhibits parental care (Winn 1958a; Pyron 1995; Zhou et al. 2015). Competition between males for females can be intense, and spawning between a single female and multiple males is common (Pyron 1995). Male *E. spectabile* in breeding condition express vivid bluish and reddish colors (Page 1983). However, female *E. spectabile* show no preference for larger or more colorful males in dichotomous trials (Pyron 1995). Thus, male coloration may instead play a signaling role in male–male competition.

## Materials and Methods

Adult *E. spectabile* were collected from Cottonwood Creek (Cumberland Co., IL) via kick‐seine in April and May 2011, during the breeding season. Males were housed in groups of four in 76‐L aquariums (bottom dimensions 76 × 30 cm) where behavioral observations took place; the aquariums contained gravel substrate and a sponge filter, which was removed for experimental observations. The males varied in standard length (mean 44.1 ± 0.46 mm SE; range 37–55 mm) and coloration. Females were separately housed in group tanks. The fish were maintained at a temperature of 20°C and a 14:10 light:dark cycle and were fed frozen bloodworms (chironomid larvae) daily.

For individual identification, each male within a tank was marked with subcutaneously injected yellow Visible Implant Elastomer (VIE; Northwest Marine Technology, Shaw Island, WA, USA) in one of the four locations (left side of first dorsal fin, right side of first dorsal fin, left side of second dorsal fin, and right side of second dorsal fin). VIE is widely used in ichthyological research for individual identification and has not been known to affect behavior (Croft et al. 2004, 2005; Leblond and Reebs 2006; Weston and Johnson 2008; Phillips and Fries 2009). The males were allowed to recover from VIE injection overnight before behavioral trials began.

### Experimental observations

Behavioral trials began with the introduction of a female to an established set of four males, who were allowed to freely compete for spawning opportunities. Following a 5‐min acclimation period, fish behaviors were observed for 1 h. If the female failed to spawn within 10 min, then the trial was discarded and the set of males was tested with another female on a subsequent day. Fish were excluded from further testing once spawning occurred; thus, each of the 16 completed trials involved a different set of males and female (total *n* = 64 males, 16 females). All trials took place between one and three days postcapture.

Three behaviors were recorded for each male: (1) the number of nosedigs attended by the male, defined by his being within one body length of the performing female (if more than one male attended, each was scored as an equal fraction of 1), (2) the number of spawning events in which the male was the first to participate, hereafter termed the “primary” male, and (3) the number of spawning events in which the male participated as a sneaker, that is, subsequent to the primary male.

### Quantification of male coloration

After experimental trials, we measured male coloration following the methods described in Zhou et al. (2014). Briefly, fish were anesthetized in 0.03% tricaine methanesulfonate (MS‐222), which maximizes color expression in darters (Gumm and Mendelson 2011). The males were then photographed under standard fluorescent lighting against a white background with a Nikon Coolpix 8700 (Nikon, Melville, NY, USA) digital camera. A ColorChecker chart (X‐Rite, Grand Rapids, MI, USA) was included in the photograph to allow correction for variation in ambient lighting, using the inCamera plug‐in (version 4.0.1; PictoColor Software, Burnsville, MN, USA) for Photoshop CS4 (Adobe Systems, San Jose, CA, USA).

**Figure 1:**
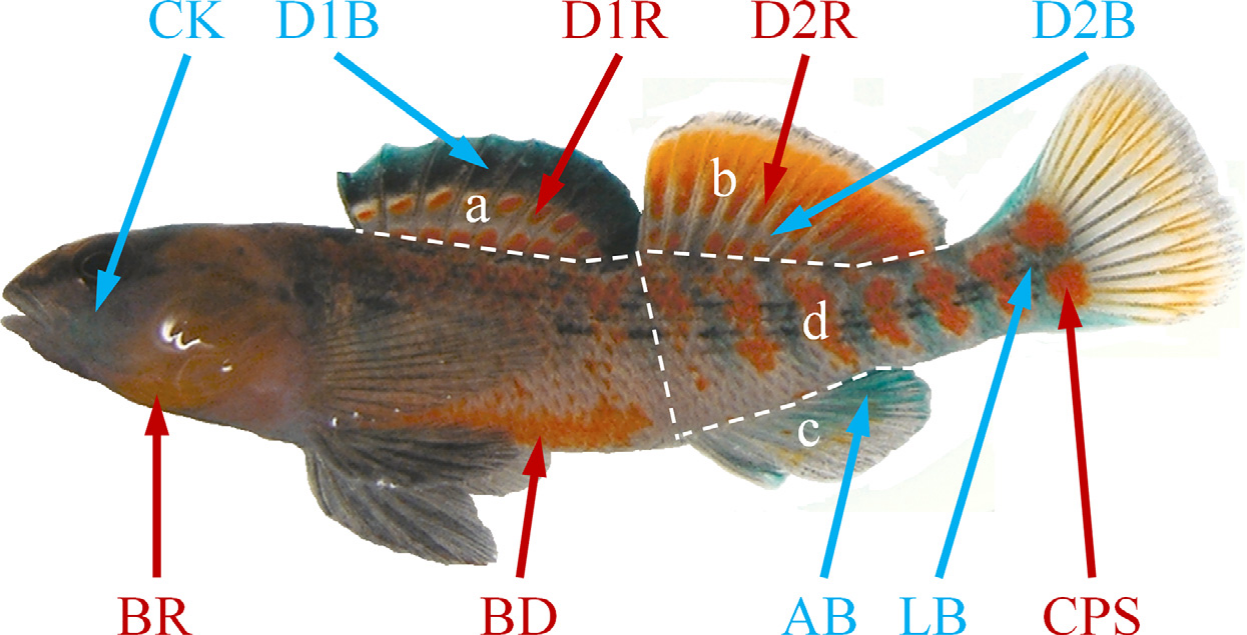
Example male *Etheostoma spectabile* showing color traits measured (uppercase labels, abbreviations given in text) and fish regions used for blue/red area measurement (a – first dorsal fin, b – second dorsal fin, c – anal fin, and d – caudal region).

Ten male color traits distributed across the body and fins were measured (Fig. 1). Five traits were categorized as “blue”: cheek (CK), first dorsal fin blue (D1B), second dorsal fin blue (D2B), anal fin blue (AB), and lateral bar (LB); and five as “red”: branchiostegal rays (BR), first dorsal fin red (D1R), second dorsal fin red (D2R), caudal peduncle spot (CPS), and abdomen (BD). RGB values were measured from each trait using the Photoshop eyedropper tool set to average from a 3 × 3 pixel square; each trait was sampled three times and the average values used. The RGB color space describes colors as an additive mixture of red, green, and blue (range 0–255). We converted the RGB values to a luminance channel R*+*G+B and two color channels (R − G)/(R + G) and (G − B)/(G + B), hereafter referred to as the red–green difference (R − G) and the green–blue difference (G − B) channels. A positive R − G value indicates a stronger red component in the color, whereas a negative R − G value indicates a stronger green component; correspondingly, a positive G − B value indicates a stronger green component in the color, whereas a negative G − B value indicates a stronger blue component.

We also quantified the proportional area of blue and red coloration on four regions of the fish: first dorsal fin (D1B area and D1R area), second dorsal fin (D2B area and D2R area), anal fin (AB area), and caudal region (CB area and CR area). Caudal region was delimited by a straight line drawn between the origins of the second dorsal and anal fins, and included the entire caudal fin (Fig. 1). The photographs were processed to isolate areas of blue and red using the Threshold Colour plug‐in (version 1.10, G. Landini) in ImageJ (version 1.43u, Wayne Rasband, National Institutes of Health, Bethesda, MD, USA). Processing took place in the CIE *Lab* color space, which describes colors in terms of L* (lightness), a* (red/green), and b* (blue/yellow). Blue areas were isolated by setting L* to exclude colors above 200/255 and b* to exclude colors above 130/255. Red areas were isolated by setting L* to exclude colors above 200/255 and a* to exclude colors below 125/255. The total area of each fish region was obtained via tracing with the ImageJ polygonal selection tool.

### Data analysis

**Figure 2:**
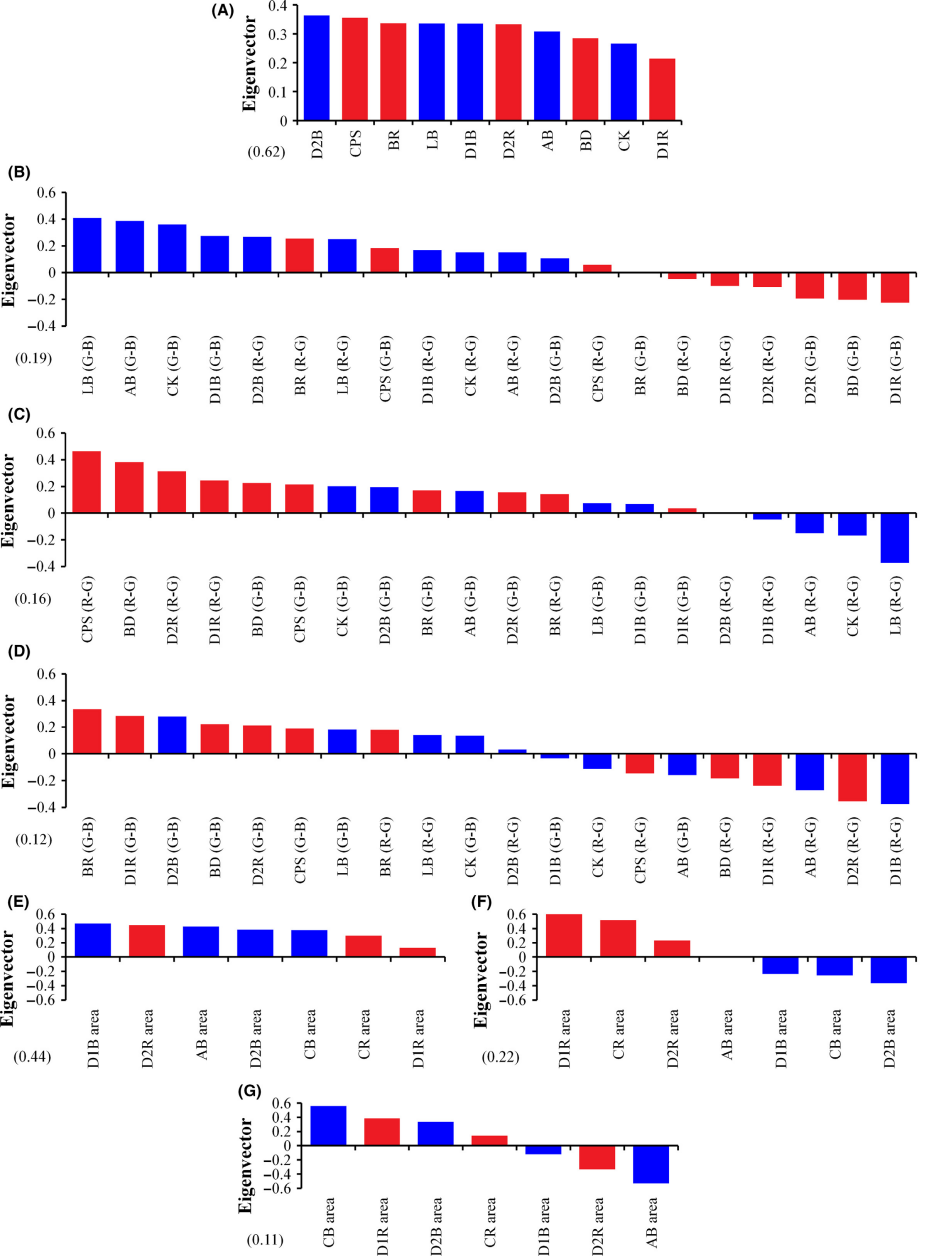
Eigenvector loadings, ordered by size, on variables included in principal component analyses, showing (A) luminance PC1, (B) color PC1, (C) color PC2, (D) color PC3, (E) blue/red area PC1, (F) blue/red area PC2, and (G) blue/red area PC3. Trait abbreviations are as given in text. Numbers in parentheses indicate the proportion of total variation accounted for by the principal component.

All statistical analyses were performed in SAS (version 9.3; SAS Institute, Cary, NC, USA). Our goal was to determine which male traits (size, luminance, color, blue/red area) were most tightly correlated with male mating success. To do this, we first used principal component analyses (PCA) to synthesize variation in luminance, color, and blue/red area across all traits (results of each PCA are given in Fig. 2; see Zhou et al. 2014 for a similar approach). Principal components that accounted for >10% of the total variation were included in subsequent analyses.

Correlates of male competitive ability and reproductive success were examined using logistic regression (GLIMMIX procedure). We analyzed the proportion of nosedigs attended, the proportion of spawning events attended as the primary, and the proportion of spawning events attended as a sneaker by each male of the total for his trial. For each analysis, the explanatory variables tested were the PC scores for luminance PC1, color PC1–PC3, and blue/red area PC1–PC3; these variables were all adjusted to be relative to their trial means. Experimental trial (*n* = 16) was set as a random effect, and we also included residuals as a random effect to scale for over‐ or underdispersion.

As several of the principal components analyzed were correlated with standard length (Table 1), we also performed the three analyses above after size‐adjusting the raw PC scores by regressing each principal component against standard length (REG procedure) and taking the residuals; standard length was then included in the model as a separate effect. Again, these variables were adjusted to be relative to the trial means.

**Table 1:** Correlations between male coloration and standard length

Principal component	β	df	*F*	*P*
Luminance PC1	−0.34	1,62	21.78	<0.01
Color PC1	−0.08	1,62	1.31	0.26
Color PC2	0.23	1,62	19.09	<0.01
Color PC3	−0.22	1,62	24.57	<0.01
Blue/red area PC1	0.33	1,62	56.42	<0.01
Blue/red area PC2	−0.01	1,62	0.05	0.82
Blue/red area PC3	−0.03	1,62	1.23	0.27

## Results

Average male size varied among trials (range 39.5–49.9 mm), but this did not alter the pattern of behavior. Invariably, at least one male attempted to guard the female, and male–male competition was intense. Males confronted one another by flaring their fins, chasing, and biting. Nosedig attendance was positively correlated with spawning as the primary male (β = 0.91, *F*_1,47_ = 333.42, *P* < 0.01) and negatively correlated with spawning as a sneaker (β = −0.27, *F*_1,44_ = 3.74, *P* = 0.06). Therefore, competitively superior males were better able to arrive first at spawning events while competitively inferior males were forced to act more as sneakers.

**Figure 3:**
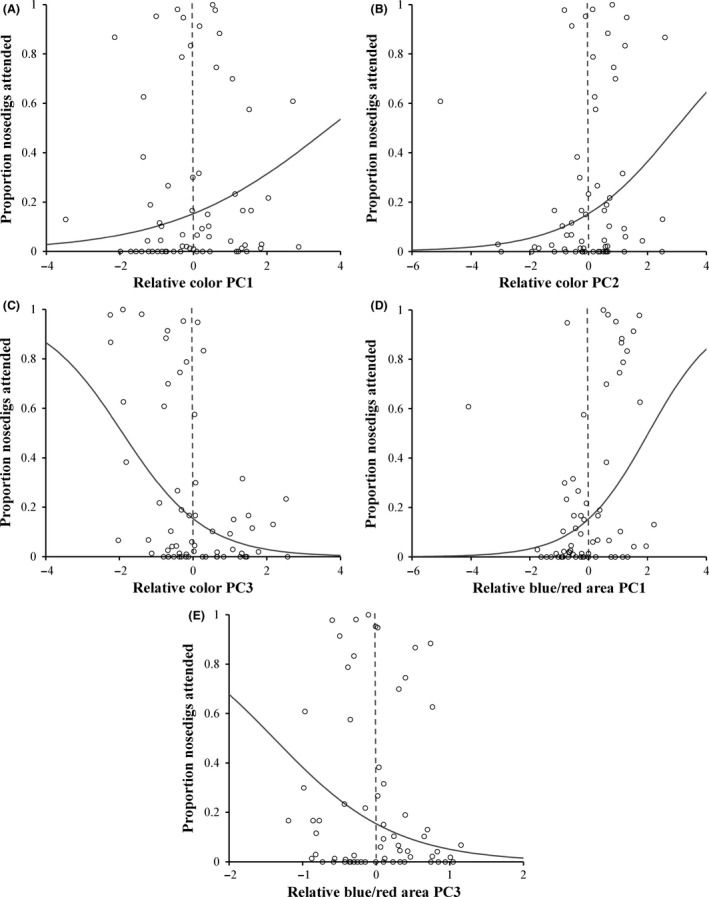
Relationships between nosedig attendance, a proxy for male competitive ability, and (A–E) components of male coloration, relative to the tank means. Each point represents an individual male.

Multiple aspects of male color and blue/red area, but not luminance, were associated with competitive and reproductive success (Table 2, Fig. 3). The first and second components of color mostly described variation in the G − B and R − G channels, respectively, with blue and red traits generally bearing opposite signs (Fig. 2A,B). Both components correlated positively with nosedig attendance and PC1 maintained this correlation with spawning as the primary male (Table 2, Fig. 3A,B). Thus, more successful males tended to be greener (lower R − G and higher G − B values) and redder (higher R − G and lower G − B values). The effects of PC1 and PC2 became nonsignificant after size adjustment (Table 2). PC3 had a larger effect than PC1 or PC2 and was consistently negatively correlated with nosedig attendance and spawning as the primary, regardless of size adjustment (Table 2, Fig. 3C). It described a more complex pattern of color variation (Fig. 2C). The red trait loadings suggest that successful males had redder dorsal fins, CPS, and abdomen (higher R − G and lower G − B values), and less orange branchiostegal rays (lower R − G and G − B values). The blue trait loadings suggest that successful males tended to have bluer first dorsal fins and LBs (lower R − G and G − B values), less blue second dorsal and anal fins (higher R − G and G − B values), and less green cheeks (higher R − G and lower G − B values).

**Table 2:** Effects of male coloration and body size on competition and spawning

Effect	β	df	*F*	*P*	After size adjustment			
					β	df	*F*	*P*
Nosedig attendance								
Luminance PC1	−0.01	1,41	0.01	0.91	−0.10	1,40	0.46	0.50
Color PC1	0.46	1,41	41.34	<0.01	0.37	1,40	1.11	0.30
Color PC2	0.58	1,41	43.28	<0.01	0.13	1,40	0.43	0.52
Color PC3	−0.89	1,41	117.95	<0.01	−0.78	1,40	5.02	0.03
Blue/red area PC1	0.84	1,41	44.29	<0.01	0.63	1,40	2.05	0.16
Blue/red area PC2	0.07	1,41	1.25	0.27	0.02	1,40	0	0.94
Blue/red area PC3	−1.23	1,41	62.95	<0.01	−0.80	1,40	2.24	0.14
Standard length						1,40	13.43	<0.01
Spawning as primary								
Luminance PC1	0.03	1,41	0.02	0.90	0.03	1,40	2.00	0.16
Color PC1	0.32	1,41	5.06	0.03	0.13	1,40	0.55	0.46
Color PC2	0.24	1,41	1.69	0.20	−0.01	1,40	0.02	0.88
Color PC3	−0.58	1,41	12.49	<0.01	−0.52	1,40	8.09	<0.01
Blue/red area PC1	1.39	1,41	30.68	<0.01	1.20	1,40	4.76	0.04
Blue/red area PC2	0.03	1,41	0.06	0.81	0.03	1,40	0.35	0.56
Blue/red area PC3	−1.09	1,41	15.93	<0.01	−0.93	1,40	4.29	0.04
Standard length						1,40	11.10	<0.01
Spawning as sneaker								
Luminance PC1	−0.18	1,38	0.52	0.48	0.14	1,37	0.16	0.70
Color PC1	0.48	1,38	7.75	0.01	0.27	1,37	0.43	0.51
Color PC2	0.25	1,38	2.45	0.13	0.24	1,37	0.03	0.86
Color PC3	0.01	1,38	0	0.95	0.00	1,37	0.03	0.86
Blue/red area PC1	−0.63	1,38	6.14	0.02	−0.21	1,37	0.02	0.90
Blue/red area PC2	−0.39	1,38	5.82	0.02	−0.35	1,37	1.83	0.18
Blue/red area PC3	0.23	1,38	0.70	0.41	0.17	1,37	0.03	0.85
Standard length						1,37	3.79	0.06

The first and third principal components of blue/red area were positively and negatively correlated, respectively, with nosedig attendance and spawning as the primary male (Fig. 3D,E). These effects remained significant after adjusting for size (Table 2). PC1 loaded positively onto all traits (Fig. 2E), indicating that males with proportionally greater amounts of blue and red were more successful. PC3 loaded most positively onto caudal region blue area and most negatively onto anal fin blue area (Fig. 2G). Therefore, more successful males tended to have greater amounts of blue on the anal fin and smaller amounts of blue on the body.

Sneaking behavior was also correlated with some aspects of color and blue/red area, although these effects were not significant after adjusting for size (Table 2). Males that spawned more as a sneaker tended to be greener (lower R − G and higher G − B values) and had proportionally lesser amounts of color (lower scores on area PC1), red in particular (lower scores on area PC2, which loaded positively onto red traits and negatively onto blue traits).

**Figure 4:**
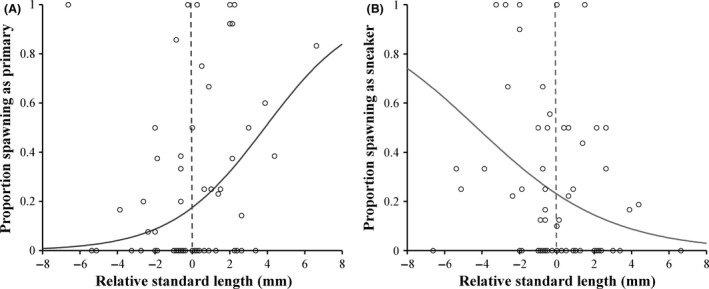
Relationships between relative body size and (A) spawning as the primary male and (B) spawning as a sneaker male. Each point represents an individual male.

Body size was strongly related to competitive ability and spawning behavior. Males that were relatively larger within their tank attended a greater proportion of nosedigs and consequently spawned more as the primary male (Table 2, Fig. 4A). Relatively smaller males tended to spawn more as sneakers, although the effect was not significant (Table 2, Fig. 4B).

## Discussion

Male–male competition is likely an agent of sexual selection in *E. spectabile*, with both body size and male coloration influencing competitive ability. We observed that male *E. spectabile* competed vigorously both to gain access to the female and to deny access to other males. Female *E. spectabile* do not exhibit preferences for large versus small males or bright versus dull males, either in association time or in latency to spawning (Pyron 1995). In lieu of overt mate choice, female *E. spectabile* appear to perform nosedigs in proportion to the amount of male pursuit (Zhou et al. 2015). Nosedig attendance thus represents a proxy metric for male competitive ability in our study, as males that are better able to keep rivals at bay and spend more time near the female should also receive a commensurately greater number of nosedigs.

Males that were competitively superior correspondingly enjoyed more opportunities to spawn as the primary male. However, sneaking behavior was common, as is the case in nature due to high population density (Pyron 1995). In egg‐burying darters such as *E. spectabile* and the closely related *Etheostoma caeruleum* Storer, 1845, only a small number of eggs are released per spawning bout (Winn 1958a; Fuller 1991; pers. obs.); thus, the participation of one or more sneaker males may entail substantial losses of paternity to the primary male. If so, this could drive strong selection for traits that enable males to monopolize access to females, that is, keeping all rivals far enough away that they cannot reach the female in the few seconds before she finishes spawning. Conspicuous visual signals may be beneficial under such conditions as they enable males to more effectively warn off potential rivals (Andersson 1994). A link between competition and coloration has been experimentally demonstrated in the Pecos pupfish (*Cyprinodon pecosensis*) Echelle and Echelle, 1978, wherein the expression of breeding coloration increased in males kept in view of other males (Kodric‐Brown 1996), and in the three‐spined stickleback (*Gasterosteus aculeatus*) Linnaeus, 1758, wherein the size of the red color patch in males increased or decreased following intrasexual contests in accordance with the outcome (Candolin 1999).

To our knowledge, our data represent the first quantitative evidence that male coloration is subject to sexual selection in darters. Both the quantity (blue/red area PC scores) and quality (color PC scores) of male coloration appear to predict spawning success. Males that expressed a relatively greater amount of blue and red coloration, especially on the anal fin, were more successful competitively and reproductively. The relationship between color quality and male reproductive success was more complex: the red components of male coloration varied in a mostly consistent manner, in that males with redder fins and bodies attended more nosedigs and spawned more as the primary male. On the other hand, more successful males were bluer on the first dorsal fin and body, and less blue on the second dorsal and anal fins. Curiously, Zhou et al. (2014) found in *E. spectabile* and *E. caeruleum* that variation in red traits was more diverse than blue traits at the across‐species and across‐population levels. One possible explanation is that there are local selective forces that act consistently on red color within populations but lead to divergence between populations.

The signaling role of male coloration in *E. spectabile* must be considered with respect to the effect of body size, as many aspects of coloration vary consistently across males of different sizes (Zhou et al. 2014). Larger male *E. spectabile* were better able to guard the females and as a result spawned more as the primary male. The advantage of larger body size in intraspecific contests has been widely documented among animal taxa, including in a variety of teleost species (e.g., Rowland 1989; Quinn and Foote 1994; Moretz 2003; Thünken et al. 2011). Many species with strong male–male competition have evolved male color signals that advertise physical attributes, that is, “badges of status”, allowing males to avoid the costs of entering pointless contests with mismatched opponents (Rowher 1975; Maynard Smith and Parker 1976; Maynard Smith 1982). Across diverse species such as house sparrows (*Passer domesticus*) (Linnaeus, 1758) (Møller 1987), the paper wasp *Polistes dominulus* (Tibbetts and Dale 2004), and Iberian rock lizards (*Lacerta monticola monticola*) Boulenger, 1905 (Martín and López 2007), direct aggression between males increases with increasing badge similarity. Darter male colors may function similarly: confrontations between male *E. spectabile* begin with visual fin flaring displays that may or may not escalate to physical attacks (Zhou et al. 2015). We found that correlations between male coloration and reproductive success occurred mostly in the same directions as the correlations between coloration and body size; furthermore, we found the same general relationship between male coloration and reproductive success before and after adjusting for standard length. These results support the notion that color is the more proximate visual signal during male–male contests: for a given size of male, the more successful individuals are those that express coloration further in the direction consistent with larger size.

Red color traits may be particular targets of intrasexual selection in darters, as the ability to perceive long‐wavelength colors seems to be necessary to elicit conspecific male aggression in *E. spectabile* (Zhou et al. 2015). Reddish coloration in darters results from carotenoid pigments (Porter et al. 2002; Zhou et al. 2014); a large body of research supports the hypothesis that carotenoid pigments represent “honest” signals of male quality, as they must be acquired from dietary sources (Candolin 2000; Grether et al. 2001; Hill et al. 2002; Navara and Hill 2003; Griggio et al. 2007). For example, the size and intensity of carotenoid‐based epaulet ornaments in male red‐shouldered widowbirds (*Euplectes ardens*) (Boddaert, 1783) predicts fighting ability (Pryke et al. 2001; Pryke and Andersson 2003). Similarly, various aspects of the carotenoid‐based coloration on the frill of the frillneck lizard (*Chlamydosaurus kingii*) Gray, 1825 correlate with competitive ability (Hamilton et al. 2013). In *E. caeruleum*, the spectral characteristics of carotenoid‐based reddish coloration are correlated with parasite load (Ciccotto et al. 2014). It is therefore plausible that red coloration may be especially important when rival male *E. spectabile* are evaluating one another.

In the context of male–male contests, the consistent pattern of variation observed by different patches of red coloration across the body and dorsal fins of male *E. spectabile* may also represent a case of redundant signaling, that is, multiple signals that together allow males to more thoroughly assess the condition of rival males. Redundant signals are predicted to be more prevalent in aggregating species due to a lowered cost of making assessments (Møller and Pomiankowski 1993; Hebets and Papaj 2005). During the spawning season, *E. spectabile* of both sexes congregate in areas with appropriate substrate, and the high number of encounters between males may favor redundant signaling so as to avoid the costs of unneeded aggression.

Female‐guarding behavior was observed in all trials irrespective of the mean male size within the tank; therefore, sneaking does not appear to be a phenotypically distinct strategy in *E. spectabile* as in some other teleost species (Oliveira et al. 2005), but rather a plastic behavioral response to relative competitive inferiority. Given that sneaking is an opportunistic act, our finding that male coloration correlates weakly, if at all, with sneaking behavior is not surprising.

A persistent complication in the study of sexually selected traits is that such traits are often involved in both attracting mates and deterring rivals, resulting in an interaction of intersexual and intrasexual selective forces that may or may not be congruent (Moore 1990; Berglund et al. 1996; Kodric‐Brown 1996; Hunt et al. 2009). Competition among males for access to females appears to have played a central role in the evolution of sexually dimorphic coloration in *E. spectabile*, and likely also in other darter species such as *E. caeruleum* that share the same mating system (Winn 1958b). Given that female *E. spectabile* do not appear to exhibit mating preferences (Pyron 1995), male breeding coloration in this species may be solely attributable to intrasexual competition. Sexual selection processes in darters may therefore illustrate the potential of male–male contests to promote the evolution of sexual ornaments.

## Conflict of Interest

None declared.
